# An economic evaluation alongside a randomized controlled trial evaluating an individually tailored lifestyle intervention compared with usual care in people with Familial Hypercholesterolemia

**DOI:** 10.1186/s13104-015-1282-x

**Published:** 2015-07-29

**Authors:** Karen Broekhuizen, Marieke F van Wier, Lando L J Koppes, Johannes Brug, Willem van Mechelen, Judith E Bosmans, Mireille N M van Poppel

**Affiliations:** Department of Public and Occupational Health, EMGO+ Institute for Health and Care Research, VU University Medical Center, Amsterdam, The Netherlands; Department of Health Economics and Health Technology Assessment, Faculty of Earth and Life Sciences, Institute of Health Sciences, VU University, Amsterdam, The Netherlands; TNO, Division Work and Employment, Hoofddorp, The Netherlands; Department of Epidemiology and Biostatistics, EMGO+ Institute for Health and Care Research, VU University Medical Center, Amsterdam, The Netherlands

**Keywords:** Cost-benefit analysis, Counselling, Cardiovascular disease, Cholesterol, Sedentary lifestyle

## Abstract

**Background:**

Cost-effectiveness analyses provide insight in the use of lifestyle interventions. To evaluate the cost-effectiveness of a lifestyle intervention compared to usual care in people with Familial Hypercholesterolemia, 340 people with FH were randomized to the intervention or control group. LDL cholesterol, quality of life and costs were measured at 0 and 12 months. Cost-effectiveness analyses were performed from a healthcare perspective using bootstrapping techniques.

**Results:**

Non-significant decreases in LDL cholesterol and quality of life were found. The mean between-group difference in costs was €−237 (95% CI −1,386 to 130). The incremental cost-effectiveness ratios were 1,729 per 1 mmol/l LDL cholesterol and 145,899 per QALY gained. Assumed that the small non-significant decrease in LDL cholesterol is attributed to the intervention, the probability of cost-effectiveness of the intervention compared to usual care was 91% per 1 mmol/l LDL cholesterol reduction and 75% per QALY gained at a ceiling ratio of €20,000.

**Conclusions:**

The intervention is not cost-effective.

Trial registration: NTR1899, date 07-07-2009.

## Background

In the Netherlands, approximately one in every 500 people is affected with Familial Hypercholesterolemia (FH) [1], which is a genetic disorder of the lipoprotein metabolism, associated with elevated plasma concentrations of LDL-C [2]. Elevated serum LDL-C and FH are associated with an increased risk of early cardiovascular disease (CVD) [3]. Since 1994, already 23,668 of the estimated 40,000 mutation carriers have been found and genetically diagnosed through the cascade screening program of the Dutch Foundation for Tracing Hereditary Hypercholesterolemia (in Dutch: StOEH) [4].

CVD is a major contributor to the global burden of disease, as it decreases quality of life and accounts for 20% of disability-adjusted life years (DALYs) lost in developed countries [5]. CVD also constitutes a large economic burden, as approximately 10% of the European health budget is spent on CVD [6]. Moreover, productivity losses due to premature death and illness of CVD patients of working age and costs due to informal care for people with CVD also contribute greatly to the societal economic burden (21% of the total costs of CVD) [6]. Results of primary prevention trials in high-risk persons and secondary prevention trials in CVD patients show that substantial reductions in CVD risk can be achieved through lifestyle changes [7–9]. Given the burden of CVD and the limited resources available for health care, information on the cost-effectiveness of available intervention strategies to reduce CVD risk is important. The aim of this study is to assess the cost-effectiveness and the cost-utility of an individually tailored lifestyle intervention compared with usual care in people with FH after 12 months from a health care perspective.

## Methods

### Design of the study

An economic evaluation was conducted from a healthcare perspective alongside a randomized controlled trial (RCT). Details on the design of the project and the intervention content have been published elsewhere [10]. The study design and informed consent procedure were approved by the Medical Ethics Committee of the VU University Medical Center and all participants provided written informed consent. The trial has been registered at dutchtrialregister.nl as NTR1899.

### Study population and setting

Participants diagnosed with the heterozygous type of FH from January 1st 2007 to April 15th 2009, aged 18–70 years and with a LDL-C level >75th percentile (age and gender specific) were recruited from the national cascade screening program of the StOEH [4]. Access to internet, sufficient fluency in Dutch and residency <150 km radius from Amsterdam were additional eligibility criteria. Conservatively, it was expected that the primary outcome LDL-C could be lowered by 35%. With an alpha of 0.05, 200 participants in the intervention and 200 in the control group and an expected drop-out of 20%, power to statistically detect an intervention effect of 9% was 90%. Participants were randomly assigned to either the control group (n = 159) or the intervention group (n = 181) through a stratified computerized blinded randomisation procedure using Microsoft© Office Access 2003 software. Participants were stratified according to cholesterol lowering medication use (yes/no), assuming that medication use implicates treatment by a general practitioner and/or medical specialist, who could have already given advice on lifestyle behavior. In addition, we expected that the potential decrease in LDL-C because of the intervention would be smaller if a participant already used medication. Family members from the same household were clustered and subsequently randomized as a cluster to prevent contamination due to spill over of communication about the intervention among family members.

### Intervention and control

The intervention consisted of a combination of tailored web-based advice (*PRO*-FIT*advice) and one face-to-face counselling session complemented with telephone booster sessions (*PRO*-FIT*coach) [10]. The goal of the intervention was to improve awareness of the CVD risk, by increasing knowledge about CVD risk based on current lifestyle behavior, cues to action and change in risk perception, and to lower LDL-C levels and adopt and maintain a healthier lifestyle, regarding physical activity, saturated fat intake, fruit and vegetables intake, smoking and compliance to statin therapy [10].

Briefly, participants were encouraged to visit a web link referring to the project website, where generic CVD risk information was presented, containing information on CVD risk behaviors and their contribution to overall CVD risk, as well as information on the changeability of these behaviors and cues on how to change behaviors. Thereafter, participants could log on to a personal *PRO*-FIT*advice account, consisting of six advice modules on smoking, physical activity, saturated fat intake, fruit intake, vegetables intake and compliance to statin therapy. On-screen personalized feedback was tailored to personal performance level (current lifestyle behavior), awareness of one’s own performance, as well as personal motivation to change, outcome expectations, attitude and self-efficacy.

Subsequently, one face-to-face counselling session was provided to each participant by a lifestyle coach at the participants’ home with a duration of 45 min. The assessment(s) and advice(s) within the participant’s personal *PRO*-FIT*advice account were discussed, and ambivalence and barriers related to the recommended behavior changes were explored using Motivational Interviewing (MI) techniques [11]. In the following 9 months, the lifestyle coach offered one to five booster telephone sessions of 15 min per participant, to encourage the participant’s behavioral changes and to provide further brief MI to encourage the planned behavioral changes.

The control group received care as usual, which means that they received no extra intervention besides the care they already received: at least one visit to the general practitioner and/or medical specialist a year and the use of cholesterol-lowering medication (approximately 70% of the participants).

### Study measures

#### Clinical outcomes

LDL-C was measured at baseline and 12 months with fasting finger stick samples analysed on a Cholestech LDX desktop analyser (Cholestech, Hayward, USA) [10]. For the cost-utility analysis, the EuroQol-5D (EQ-5D) was used to assess quality of life at baseline and at 12 months [12]. To estimate the utility of health states described by the participants, the Dutch tariff was used [13]. Quality adjusted life years (QALYs) were calculated by multiplying the utilities with the amount of time a participant spent in a particular health state. Transitions between health states were linearly interpolated.

### Cost measures

Data were collected from a healthcare perspective, i.e. only healthcare-related costs were included in the economic evaluation. Prices were adjusted for the year 2010, the year in which most data were collected, using consumer price indices [14].

Information on healthcare utilization and prescribed medication associated with FH and/or CVD was obtained through a 12-month retrospective questionnaire. Healthcare utilization consisted of costs of primary care (including general practitioner and therapist care) and secondary care (including medical specialist care and hospitalization associated with FH and/or CVD), and were valued with Dutch standard costs [15]. If these were not available, prices according to professional organizations were used. The costs of prescribed medication were calculated using prices charged by the Royal Dutch Society for Pharmacy [16].

Intervention costs were estimated using a bottom-up micro-costing approach, i.e. detailed data were collected regarding the quantity of resources consumed per patient as well as their unit prices. Costing was based on the assumption that the intervention would be implemented for a 5-year period by an academic medical center. According to StOEH data, approximately 2,700 people would be eligible and willing to participate during this period [4]. Consequently, five lifestyle coaches would be needed for the coaching component of the intervention. Variable costs per participant depended on the number of counselling sessions received and were calculated using annual salaries of the lifestyle coaches with added taxes and benefits. Intervention costs additionally included costs of the development and implementation of materials, training and supervision of the lifestyle coaches, and the development and implementation of the PRO-FIT*advice web-environment.

### Statistical analyses

Missing healthcare costs, QALY data and LDL-C levels were multiply imputed in SPSS 17 creating ten different data sets [17–19]. Data were imputed separately for the intervention and control group. The imputational model included important demographics and prognostic variables associated with the missing data: age, gender, LDL-C levels and body mass index (BMI) at baseline and follow-up, intervention costs, primary care (general practitioner and therapist) costs, secondary care (outpatient visits and hospital admission) costs and medication costs, and utilities at baseline and follow-up. Pooled estimates of effects and costs were estimated according to Rubin’s rules [20].

Main analyses were according to the intention to treat principle and based on the imputed data. Differences in baseline characteristics between the intervention and control group and between cases with missing data and cases with complete data were tested using linear and logistic regression analysis. The effects on clinical outcomes at 12 months were analysed using linear regression analyses, adjusted for baseline values. Mean cost differences between the intervention and control group were calculated for primary and secondary care, medication, and total costs. The Approximate Bootstrap Confidence algorithm with 5,000 bootstrap samples was used to estimate 95% confidence intervals surrounding the cost differences [21]. Incremental cost-effectiveness ratios (ICERs) were calculated by dividing the difference in total costs between the intervention and control group by the difference in clinical outcomes adjusted for baseline values. The ICER indicates the additional investments needed for the intervention group to gain one extra unit in health effect, i.e. 1 mmol/l LDL-C and 1 QALY, in comparison with usual care. The bootstrapped cost–effect pairs were graphically presented in a cost-effectiveness plane, to show the uncertainty around the ICER. Cost-effectiveness acceptability curves (CEACs) were also estimated. CEACs show the ‘willingness to pay’ for a unit of health effect extra (i.e. ceiling ratio) on the x-axis and the corresponding probability that the intervention is cost-effective at that ceiling ratio on the y-axis. All analyses were done in R (version 2.10.1) [22].

To assess the robustness of the results, three sensitivity analyses were performed. First, a cost-effectiveness analysis (CEA) taking only complete cases into account was conducted (CEA2). Second, a CEA was performed using the actual costs of the PRO-FIT intervention within the PRO-FIT trial (including 340 participants, two lifestyle coaches, implemented in a 1-year period) (CEA3). Third, a CEA was conducted in which the hospital admission costs were excluded from the total costs (CEA4).

## Results

### Participant flow and baseline characteristics

**Fig. 1:**
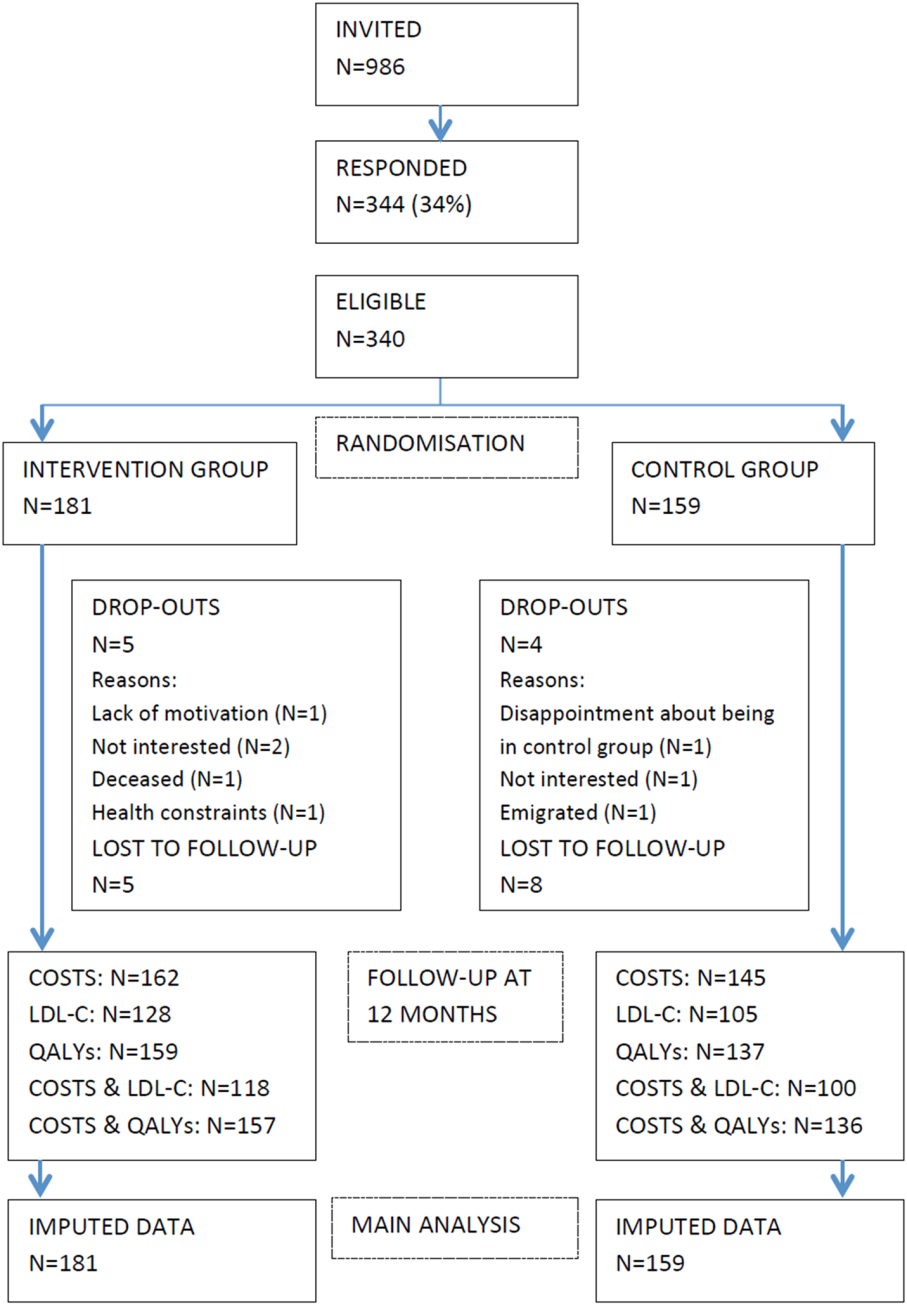
Flow of participants in the PRO-FIT project. Figure 1 shows the flow of participants from recruitment to analysis in the PRO-FIT project, conducted in 2009–2010 in the Netherlands among 340 adults with Familial Hypercholesterolemia.

Invitation letters were sent to 986 people, of whom 340 (34%) responded and participated in the trial. The participant flow is presented in Fig. 1. A small proportion of participants decided to discontinue participation or was lost to follow-up in both the intervention (5%) and control group (8%), resulting in 318 participants completing the study. The number of participants with complete follow-up data ranged from 64 to 90%. Baseline characteristics are given in Table 1. A significant difference in baseline BMI between intervention and control group was found (mean difference = −1.10; 95% CI: −2.16 to −0.05) in the imputed and complete cases dataset. As a consequence, baseline BMI values were included in all analyses of cost-effectiveness regarding LDL-C and QALYs.

**Table 1:** Baseline characteristics of people with Familial Hypercholesterolemia in control and intervention group after multiple imputation

	Control group N = 159	Intervention group N = 181
Gender, N (% female)	90 (57)	104 (57)
Mean age in years (SEM)	46.0 (1.0)	44.7 (1.0)
Mean BMI in kg/m^2^ (SEM)	*27.1* (*0.4*)	*26.0* (*0.3*)
Statin use, N (% yes)	110 (69)	123 (68)
EQ-5D utility score (SEM)^a^	0.9 (0.01)	0.9 (0.01)

Significant differences between control and intervention group (P < 0.05) are printed in italics font.

^a^Assessed by the EuroQol-5D; *N* sample size, *SD* standard deviation, *BMI* body mass index, *SEM* standard error of the mean.

### Intervention compliance

Of the 181 participants in the intervention group, 95% received a PRO-FIT*advice log on account. The remaining 5% (9 participants) explicitly reported to have no interest in using *PRO*-FIT*advice and therefore, received no log on information. Subsequently, 49% of remaining 172 participants actually logged on and completed at least one out of six advice modules. Nearly all participants (99%) received the face-to-face counselling session and on average, 4.2 telephone booster calls were conducted with 181 participants.

### Clinical outcomes

After 12 months, LDL-C had decreased in both groups and by 0.14 mmol/l more in the intervention group. The intervention group had 0.002 QALYs less than the control group. These between-group differences for LDL-C and QALYs were small and statistically non-significant (see Table 2).

**Table 2:** Pooled intervention effects on LDL-C and QALYs after 12 months

Pooled effects (pooled mean (SEM))	Control group N = 159		Intervention group N = 181		Intervention versus control
	Baseline	Follow-up	Baseline	Follow-up	Mean difference (95% CI)
LDL-C (mmol/l)	3.7 (0.1)	3.6 (0.1)	3.7 (0.1)	3.5 (0.1)	−0.14 (−0.34 to 0.07)
QALYs achieved	–	0.9 (0.01)	–	0.9 (0.01)	−0.002 (−0.02 to 0.01)

Effects were calculated after multiple imputation and with adjustment for baseline values.

*LDL-C* low-density lipoprotein cholesterol, *QALY* quality adjusted life year, *SEM* standard error of the mean; the maximum QALY that can be achieved in one year is 1 unit.

### Costs

Intervention costs are presented in Table 3 and mainly consisted of the costs of counselling (91%). Pooled mean costs and cost differences between the intervention and control group are presented in Table 4. Around one-third of total costs in both groups consisted of medication costs. Primary care costs were statistically significantly lower in the intervention group in comparison with the control group. Secondary care costs in the control group were considerably higher than in the intervention group due to one extended hospitalization in this group. However, the difference in secondary costs was not statistically significant. Overall healthcare-related costs were €237 lower in the intervention group but this difference was not statistically significant (−1,386 to 130).

**Table 3:** Overview of costs of the PRO-FIT intervention in Euros per participant

Cost category	Included resources	Cost prices per unit^a^	Costs per participant
Development			
Developmental costs of brochure and coaching logs	Content development (30 h) by junior researcher Concept development/graphic design (24 h) by graphic designer Final development (12 h) by brochure designer	€ 35.75/h € 75/h € 65/h	Є 2.80
Computer-based part of intervention, including website and application for providing computer-tailored advice	Web development (12 h) by web-developer Registration website (once) Development/adjustment tailoring application by junior researcher (216 h) Account tailoring application	€ 65/h € 53.95^§^ € 35.75/h € 3,930.25^§^	Є 5.44
Brochures, logs, website and tailoring application	Printing of brochure/coaching logs Hosting website Hosting tailoring application	€ 0.10/piece € 119.40/year^§^ € 171/year^§^	Є 1.64
Implementation based on 2,700 participants and an implementation period of 5 years			
Training of lifestyle coaches	A 3-day Motivational Interviewing workshop 5 lifestyle coaches, 3 days, 8 h/day Supervisor, 3 days, 8 h/day	€ 5,100^§^ € 38.38/h € 35.75/h	Є 3.94
Supervision of lifestyle coaches (10 meetings of 2 h each)	Meeting rooms rental costs 5 lifestyle coaches Supervisor	€ 11.50/room/h^§^ € 38.38/h € 35.75/h	Є 1.77
Counselling	1 face-to-face counselling session (45 min) by lifestyle coach 5 telephone booster sessions (15 min/session) by lifestyle coach Administrative work (25 min/participant) by lifestyle coach Travelling (82 km/participant and 1 h/participant)	€ 38.38/h € 38.38/h € 38.38/h € 0.20/km € 38.38/h	Є 147.64
Total intervention costs			Є 163.13

*hrs* hours, *mins* minutes.

^a^Salary costs were derived from the Collective Labour Agreement for Dutch Academic Medical Centers (CAO UMC) 2010 (for junior researcher, lifestyle coach and supervisor), or by price offers from web developers, graphic/brochure designers.

^§^Costing was based on invoices/price offers.

**Table 4:** Pooled mean differences in healthcare-related costs per participant in Euros between baseline and 12-months follow-up

Pooled costs [pooled mean (SEM)]	Control group	Intervention group	Mean cost difference (%CI)
PRO-FIT intervention	0	163	163 (NA)
Primary care	*86 (17)*	*44 (8)*	*−43 (−86 to −11)*
Secondary care	461 (289)	121 (51)	*−*340 (*−*1,406 to 24)
Medication^a^	284 (29)	266 (23)	*−*17 (*−*91 to 54)
Total costs	831 (297)	594 (60)	*−*237 (*−*1,386 to 130)

*SEM* standard error of the mean, *NA* not available; costs are given in 2010 Euros; mean differences were calculated after multiple imputations.

^a^Prescribed statins.

### Cost-effectiveness

**Fig. 2:**
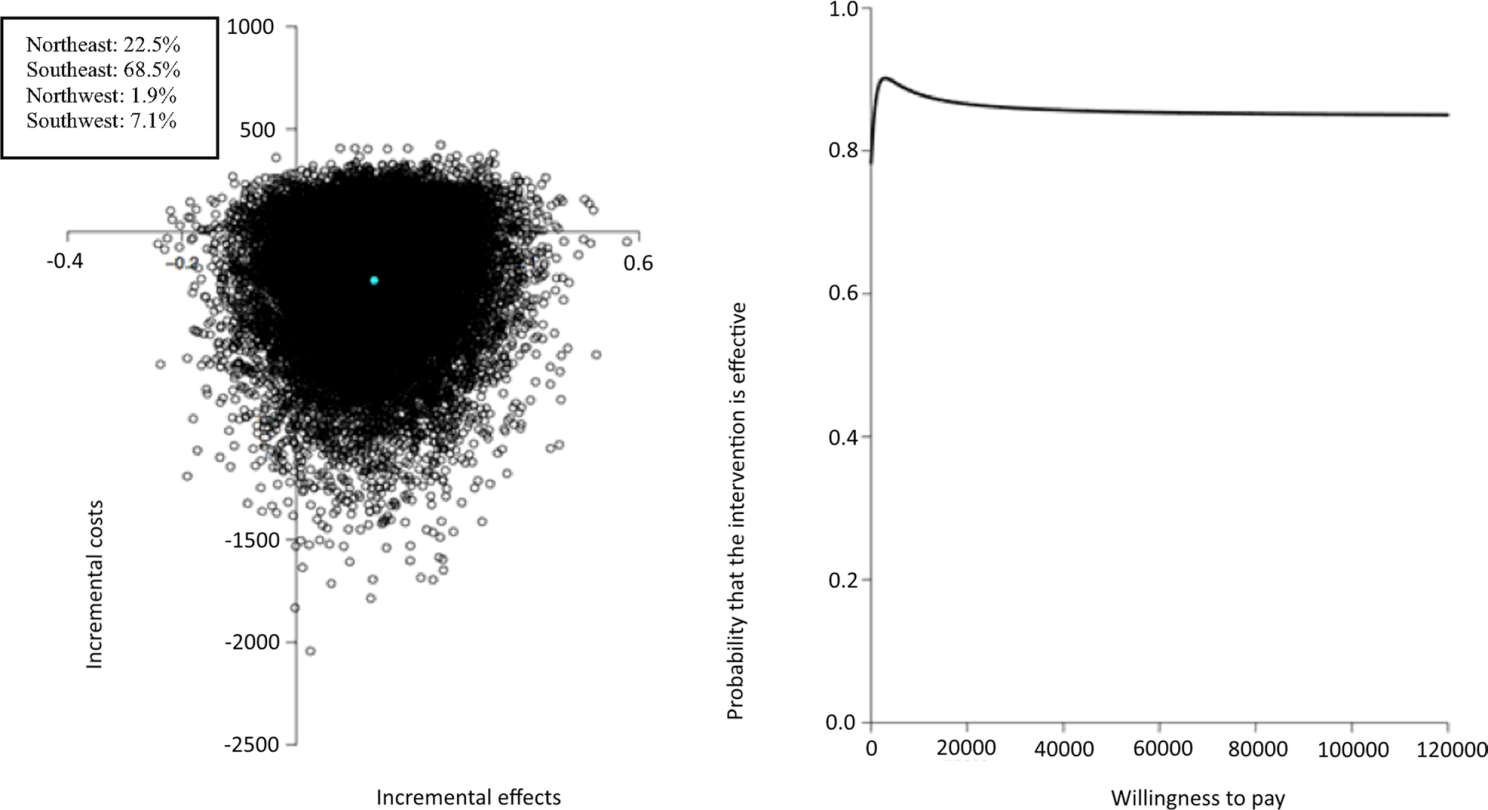
a and 2b: Pooled cost-effectiveness plane and cost-effectiveness acceptability curve for the difference in LDL-C after 12 months. Figure 2a and 2b show the pooled cost-effectiveness plane and cost-effectiveness acceptability curve for the difference in LDL-C after 12 months within the PRO-FIT project, conducted in 2009–2010 in the Netherlands among 340 adults with Familial Hypercholesterolemia.

Assuming that the non-significant difference in LDL-C between intervention and control group can be attributed to the intervention, the main analysis showed that the pooled ICER for LDL-C was €1,729 (see Table 5), indicating that a 1 mmol/l decrease in LDL-C concentration extra as a result of the PRO-FIT intervention saves €1,729, compared to usual care. The cost-effectiveness plane for LDL-C (Fig. 2a) showed that 68% of the bootstrapped cost-effectiveness pairs were located in the southeast quadrant, the quadrant in which the intervention is dominant over usual care. The CEAC curve (Fig. 2b) showed that if a decision maker is willing to pay €4,000 for 1 mmol/l LDL-C reduction, the probability that the PRO-FIT intervention is cost-effective is 93%, but thereafter reduces to a maximum of 91%.

**Table 5:** Results for cost-effectiveness and cost-utility analyses

	Sample size			Cost difference in Euros (95% CI)	Effect difference (95% CI)	ICER	Distribution cost-effectivess plane (%NE/SE/SW/NW)
	I	C					
Main analysis (CEA1)	181	159	LDL-C	−237 (−1,386 to 130)	−0.14 (−0.34 to 0.07)	1,729	22.5/68.5/7.1/1.9
	181	159	QALY	−237 (−1,386 to 130)	−0.002 (−0.02 to 0.01)	145,899	9.7/30.9/44.2/15.2
Complete case analysis (CEA2)	118	100	LDL-C	−364 (−2,030 to 238)	−0.14 (−0.37 to 0.08)	2,012	4.6/8.0/55.7/31.7
	157	136	QALY	−301 (−1,680 to 109)	−0.003 (−0.03 to 0.03)	100,347	6.5/25.4/52.5/15.6
Intervention costs as in RCT (CEA3)	181	159	LDL-C	−88 (−1,248 to 277)	−0.14 (−0.34 to 0.07)	645	39.4/51.6/5.5/3.6
	181	159	QALY	−88 (−1,248 to 277)	−0.002 (−0.02 to 0.01)	54,426	17.1/23.4/33.8/25.7
Hospital admission costs excluded (CEA4)	181	159	LDL-C	94 (−6 to 193)	−0.14 (−0.34 to 0.07)	−690	88.5/2.4/0.5/8.5
	181	159	QALY	94 (−6 to 193)	−0.002 (−0.02 to 0.01)	−33,676	38.9/1.2/1.7/58.2

### Cost-utility

The ICER of €145,899 per QALY indicates that 1 QALY lost as a result of the PRO-FIT intervention saves the healthcare sector €145,899, compared to usual care (see Table 5).

**Fig. 3:**
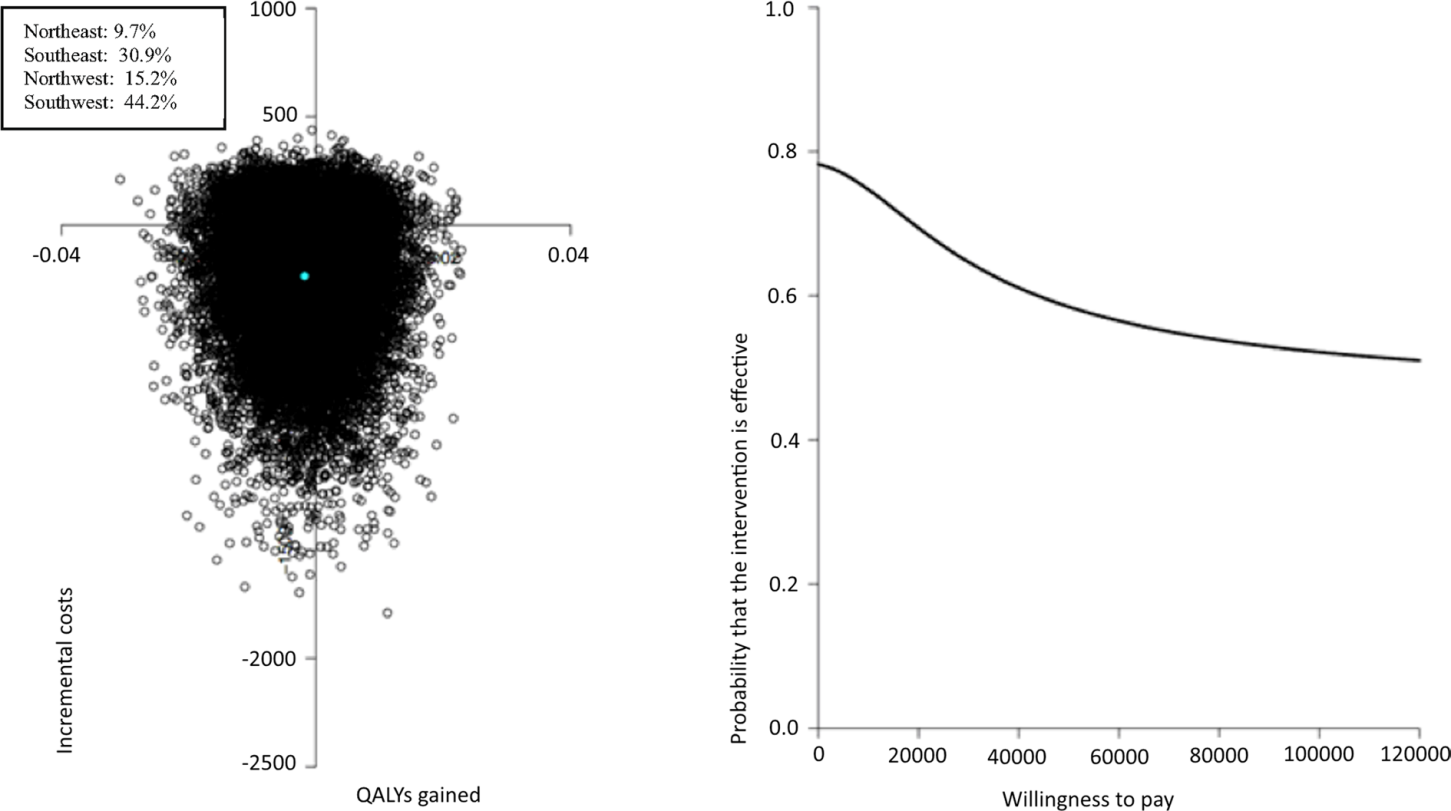
a and 3b: Pooled cost-effectiveness plane and cost-effectiveness acceptability curve for QALYs gained after 12 months. Figure 3a and 3b show the pooled cost-effectiveness plane and cost-effectiveness acceptability curve for the difference in QALYs after 12 months within the PRO-FIT project, conducted in 2009–2010 in the Netherlands among 340 adults with Familial Hypercholesterolemia.

In the cost-effectiveness plane (Fig. 3a), most cost-utility pairs (44%) were located in the southwest quadrant, the quadrant in which less QALYs are gained at lower costs in the intervention group compared with usual care. The CEAC (Fig. 3b) indicated that the probability of cost-utility of the PRO-FIT intervention compared to usual care ranged from approximately 75% at a ceiling ratio of €0 per QALY gained to 55% at a ceiling ratio of €120,000 per QALY gained.

### Sensitivity analyses

Results of the sensitivity analyses based on complete cases (CEA2) and based on the actual intervention costs of the PRO-FIT intervention (CEA3) were similar to the results from the main analyses (see Table 5). The CEA that excluded hospital admission costs led to smaller cost differences and costs were lower in the control group.

## Discussion and conclusions

The results of this study show that the PRO-FIT intervention was not cost-effective in comparison with usual care. No statistically significant differences were found in LDL-C, QALYs and health care costs after 12 months. Our study is the first to evaluate the cost-effectiveness of a lifestyle intervention compared to usual care in a FH sample. Other studies concluded that lifestyle interventions are cost-effective in reducing the long-term risk of type 2 diabetes and CVD [23]. However, our findings show no value in the addition of lifestyle advice to treatment with statins, which has already been shown to be cost-effective in people with FH [24].

All further discussion and interpretation of the present results regarding cost-effectiveness should obviously be regarded with caution, since we cannot conclude that the non-significant decrease in LDL-C and related gain in QALYs were coincidental or caused by the intervention. For the sake of this economic evaluation, the found differences compared to the usual care were regarded as real and attributable to the intervention. Having conducted a CEA for an intervention for which no evidence of effect was found as compared to usual care seems to have limited value. Though, conducting CEAs while significant effects are lacking is of great importance, e.g. for systematic reviews on the cost-effectiveness of interventions. These reviews are often hampered by a publication bias, since CEAs are generally only conducted if an intervention was significantly effective and are therefore overrepresented [25, 26]. Further, this study examines the joint distribution of costs and effects. This is relevant because even if costs and effects show no significant differences, the joint distribution could indicate that a treatment is cost-effective in comparison with control for some ceiling ratios [27]. In addition to the economic evaluation, the transparent oversight of the intervention costs and healthcare-related costs that we provided is relevant for policy-makers and future researchers planning a similar RCT.

Intervention costs were computed as if the intervention was implemented with full compliance. Taking into account the actual compliance during the trial would not lead to a substantial difference in intervention costs, as the proportion of participants that received face-to-face counselling was 99%. However, the intervention costs in this study were based on five telephone booster calls, whereas on average 4.2 were conducted during the trial. Consequently, the actual intervention costs are only slightly less (€155.46 instead of €163.13).

Secondary care costs in the control group were considerably higher than in the intervention group and this contributed most to the difference in total healthcare-related costs between the groups. Further analysis showed that this was caused by higher mean hospital admission costs associated with FH and/or CVD in the control group than in the intervention group. A sensitivity analysis excluding hospital admission costs showed that, in contrast to the main CEA analysis, costs in the intervention group were higher than in the control group, but this difference was not statistically significant and the intervention was still not considered cost-effective.

Limitations of this economic evaluation should be taken into consideration. At first, the evaluation was performed from a healthcare perspective, while Dutch guidelines recommend adapting a societal perspective. We chose this perspective since our central aim was to lower LDL-C with lifestyle changes, and no effects on productivity costs due to the intervention in the follow-up period were expected. Second, information on healthcare utilization and prescribed medication was obtained through a 12-month retrospective questionnaire. Shorter recall periods reduce the chance of recall bias, though more frequent measurements with a shorter recall period could have increased the chance of missing data, compared with one measurement with a recall period of 12 months [28]. Third, whereas intervention costs were complete, data on healthcare-related resource use and LDL-C/QALYs were missing for 36 and 14% of the participants, respectively. To account for these missing data, multiple imputation techniques were used. Multiple imputation is preferred over complete case analysis [28], since a complete-case analysis is inefficient, as the sample size is smaller and it ignores observed cost and/or effect data in the excluded participants. The advantage of using multiple imputation is that the uncertainty associated with imputing missing values is also taken into account in the pooled estimates. Overall, it is likely that the effect of lifestyle improvements is likely to lead to CVD risk reduction at the longer term (>12 months). Inclusion of more long-term follow-up measurements would clarify intervention effects on CVD risk and hard outcomes (e.g. CVD/death) and would allow a more thorough cost-effectiveness analysis.

In conclusion, an individually tailored lifestyle intervention in people with FH was not cost-effective compared to usual care. Due to the non-significant small effects found in the study, the conclusions should be regarded with caution.

## Abbreviations

FH: Familial Hypercholesterolemia
LDL-C: low-density lipoprotein cholesterol
CI: confidence interval
QALY: quality adjusted life years
DALY: disability adjusted life years
CVD: cardiovascular disorders
StOEH: Stichting Opsporing Erfelijke Hypercholesterolemie
RCT: randomized controlled trial
MI: motivational interviewing
BMI: body mass index
ICER: incremental cost-effectiveness ratio
CEAC: cost-effectiveness acceptability curve
CEA: cost-effectiveness analysis
